# Optimized Energy Harvesting, Cluster-Head Selection and Channel Allocation for IoTs in Smart Cities

**DOI:** 10.3390/s16122046

**Published:** 2016-12-02

**Authors:** Saleem Aslam, Najam Ul Hasan, Ju Wook Jang, Kyung-Geun Lee

**Affiliations:** 1 Department of Electrical Engineering, Bahria University, E-8 Naval Complex, Islamabad 44000, Pakistan; saleem.aslam@bui.edu.pk; 2 Department of Electrical and Computer Engineering, Dhofar University, Salalah 211, Oman; nulhasan@du.edu.om; 3 Department of Electronics Engineering, Sogang University, Seoul 04107, Korea; 4 Department of Information and Communication Engineering, Sejong University, Seoul 05006, Korea; kglee@sejong.ac.kr

**Keywords:** RF energy harvesing, internet of things, cognitive radio, channel scheduling, quality of service, clustering, heterogenity

## Abstract

This paper highlights three critical aspects of the internet of things (IoTs), namely (1) energy efficiency, (2) energy balancing and (3) quality of service (QoS) and presents three novel schemes for addressing these aspects. For energy efficiency, a novel radio frequency (RF) energy-harvesting scheme is presented in which each IoT device is associated with the best possible RF source in order to maximize the overall energy that the IoT devices harvest. For energy balancing, the IoT devices in close proximity are clustered together and then an IoT device with the highest residual energy is selected as a cluster head (CH) on a rotational basis. Once the CH is selected, it assigns channels to the IoT devices to report their data using a novel integer linear program (ILP)-based channel allocation scheme by satisfying their desired QoS. To evaluate the presented schemes, exhaustive simulations are carried out by varying different parameters, including the number of IoT devices, the number of harvesting sources, the distance between RF sources and IoT devices and the primary user (PU) activity of different channels. The simulation results demonstrate that our proposed schemes perform better than the existing ones.

## 1. Introduction

With the unprecedented advancements in wireless sensor networks (WSNs), the internet of things (IoTs) paradigm has emerged as one of the potential technologies for future applications and services [1]. These applications are predicted to encompass numerous fields ranging from health care to surveillance. It is also expected that these upcoming applications will revolutionize the human lifestyle by bringing ease and comfort in daily life experiences and activities [2,3]. Formally, the IoTs network can be defined as a network of physical objects, devices, vehicles and other items interconnected with different kinds of micro-controllers, equipped with transceivers and embedded with protocols for the dissemination of their sensing and control information [4]. The current state-of-the-art technologies for the IoTs network includes radio frequency identification (RFID), ulta wideband (UWB) and Zigbee, etc. [5]. However, these technologies are meant only for short-range communication, which may result in a number of isolated networks. With such a large number of isolated networks (i.e., networks that are disconnected from each other), it is impossible to realize a bigger picture of the IoTs network such as smart city. Also, these technologies usually operate in the unlicensed spectrum band, which provides very limited control over quality of service (QoS). Therefore, it is highly recommended to have a technology for the future IoT network that can provide short-range as well as long-range communication and also fulfill the QoS expectation of futuristic applications. A cognitive radio (CR) can be a viable solution in this case, as it is technology independent because it is built on the top of software-defined radio. Moreover, it can fulfill the desired QoS for IoTs by exploiting both the licensed and unlicensed spectrum. Therefore, in this paper, we propose a CR-based IoT framework. Furthermore, as IoTs are energy-constraint devices, it is desirable to make them energy efficient because in many applications, it becomes really hard to replace IoT devices or their batteries [6]. As an example, consider the deployment of IoTs in harsh environments (i.e., very high temperature or very low pressure). For such application scenarios, the radio frequency (RF) energy harvesting is the most feasible solution. Therefore, in our proposed framework, we consider that IoT devices harvest energy from ambient RF sources. In this paper, we present a novel channel allocation scheme for IoTs that considers a number of parameters, including PU activity, the QoS requirement of IoTs and their residual energies. The major contributions of this paper can be summarized as follows:

1. An IoT framework is presented with a focus on energy efficiency, energy balancing and QoS. To achieve energy efficiency, a scheme is developed to assign RF sources to IoT devices in such a way that RF devices can harvest the energy up to the maximum.
2. A clustering mechanism is introduced to cluster the IoT devices in a close proximity, and a cluster head (CH) selection strategy is proposed for that cluster. The cluster-member with the highest residual energy is selected as a CH to balance the energy consumption among the IoT devices in that cluster.
3. To fulfill the QoS requirements of IoTs, a novel integer linear program (ILP)-based channel-allocation scheme is presented to allocate the best possible channels to IoT devices during the reporting process.
4. To evaluate the performance of the proposed framework, exhaustive simulations are carried out by varying different parameters (e.g., number of channels, the number of devices, harvesting sources, etc.).

The rest of the paper is organized as follows. Section 2 presents the related work in the domain of cluster head selection, node-source association and channel allocation. Section 3 describes the system model. Section 4 describes three proposed schemes. Simulation results are presented in Section 5. Finally, Section 6 concludes the findings and sets the future directions.

## 2. Related Work

A smart city is a vision for the efficient management of resources such as water, electricity, transport and parking, the lights of roads and the parks, the supervision and monitoring of hospitals and schools, etc. The IoT network is anticipated to be the key for the realization of this kind of smart city [7]. For example, for the efficient use of water resources, the city water management department should be able to access the weather forecast information so that it can hold the process of watering plants based on the rain forecast to save a significant amount of water [8,9]. We consider that our proposed IoTs framework is an integral part of the smart city, which monitors an environment and routs the monitored data to a central place for better management of the city. IoT devices are usually characterized as energy-constraint devices, therefore, RF energy harvesting (RF-EH) is an alluring solution for addressing the energy limitations of these devices [10,11,12,13,14]. RF-EH can be classified into two types: (1) in-band harvesting and (2) out-of-band harvesting. The authors in [15,16] presents in-band harvesting schemes, whereas the schemes in [17,18] present out-of-band energy harvesting for WSNs. In [19,20], RF-EH mechanism is described for cognitive radio networks (CRNs). In [21,22,23,24,25], the authors also present the RF-EH mechanism for CRNs, but RF sources are placed differently in terms of their geometrical distributions. However, all of these schemes consider only a single RF source for the harvesting process, which lacks the efficient use of RF sources and also ignores the throughput and energy balancing aspects of the network. In addition, these schemes lack in achieving energy-balancing and QoS.

Usually, a large number of IoT devices are deployed in a close proximity to report a certain event. If all the IoT devices report their data to sink directly, this may result in the wastage of their energy. Therefore, a clustering mechanism is adopted to preserve the energy of the IoTs network. Further, the entire communication between IoT devices and sink is governed via CH. In this case, the energy of the CH may become depleted very sharply, which may limit the lifetime of the IoTs network. Therefore, a dynamic CH selection method for energy balancing is required to balance the energy among IoT devices in the IoTs network [26,27,28,29,30,31]. The authors in [27,28,29] presents a clustering mechanism for preserving the energy of WSNs, but they assume a fixed CH. Although [30,31] present a CH selection strategy for balancing the energy among nodes in WSNs, they do not consider that the sensors are harvesting energy from RF sources. Therefore, their work is not suitable for the energy-harvested IoTs network due to the dynamic energy variations in the residual energy of the IoT devices. Thus, in this paper, we present a clustering mechanism along with CH selection for energy balancing for the RF-EH IoTs network.

The IoT network consists of a massive number of devices. Each IoT device may have different requirements in terms of throughput. However, the available spectrum is very limited. Therefore, to support such a massive number of devices and to provide them their desired throughput, a highly spectral efficient technique is required to address this issue. A possible way for accomplishing this is to extend the operating frequency using cognitive radio (CR) technology [32,33,34,35]. The CR is an intelligent radio that learns, decides and reconfigures itself according to the available portion of the spectrum [36]. There are two kinds of users in CRNs: (1) primary users (PUs) and (2) CR users.

In CRNs, the CR users perform communication in an opportunistic manner on a portion of the spectrum not utilized by the PU [37,38]. Without the loss of generality, we use the same terminologies and consider the IoT devices as CR-based devices, which opportunistically access the spectrum band for their transmission. Jain et al. [38] present a channel-scheduling algorithm for RF energy-harvesting networks to reduce the outage probability of PUs and secondary users (SUs). However, this scheme uses only PUs as a harvesting source. In [39], the authors present a similar approach to [38], but they analyze the results in terms of the efficiency of RF harvesting mechanism. In [40,41,42], the authors extend the work in [39] by allowing the CR users to also harvest from other ambient RF sources in addition to PU. However, this scheme is aimed at reducing the power consumption but ignores the throughput demands of CR users. Similarly, the work of [43,44,45,46] targets the energy efficiency of CR users, but they do not consider the PU activity. Also, all of the aforementioned schemes [37,38,39,40,41,42,43,44,45,46] ignores the availability of the channels due to their lack in consideration of PU activity. To illustrate the performance gain of our proposed scheme, we compare the greedy and random schemes with the proposed RF source association and channel-sharing mechanisms [34,47]. In a random association mechanism, the regional manager node (RMN) randomly associates IoT device *i* with probability 1/N and RF source *j* with probability 1/S. Similarly, other IoT devices and RF sources are associated with the same procedure. Similarly, the greedy scheme performs association of IoT device *i* with RF source *j*, which results in the highest harvesting energy. After association, that IoT device and RF source are marked as associated and they are removed from the list. The same procedure is repeated for other IoT devices and RF sources. For channel-sharing scheme, we use the same procedure for random and greedy schemes but in the context of channel sharing.

## 3. System Model

### 3.1. Network Model

**Figure 1:**
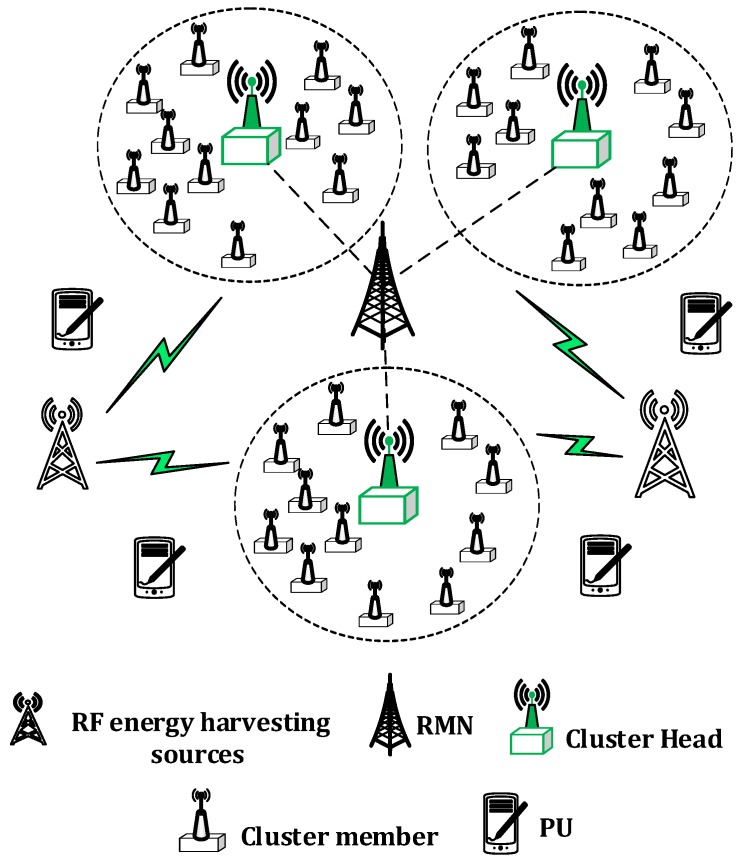
Proposed system model.

We consider a communication environment with $\bar{P}$ ambient primary RF harvesting sources, $\bar{S}$ secondary RF sources, N IoT devices, M nonoverlapping and orthogonal channels as depicted in Figure 1 . Furthermore, we consider a regional manager node (RMN), which can be thought of as an IoT device with additional energy and processing capabilities. The RMN performs an optimal association between IoT devices and RF sources to maximize the overall harvesting energy of the IoTs network (See Section 4.2 for details). We assume that RMN is perfectly synchronized with CHs and IoT devices. Furthermore, we exploit the heterogeneity of ambient RF sources in terms of operating band (e.g., 700 MHz 900 MHz, 1800 MHz and 2100 MHz) and level of the transmission power. Furthermore, the dynamic on-off activity of secondary RF sources is considered for the better realization of the harvesting environment. We also assume that IoT devices can tune themselves to different RF bands (based on the outcome of the association scheme) to cater the heterogeneity of the RF sources. To achieve energy balancing, an energy-efficient clustering scheme is proposed that groups the N IoT devices into L clusters with $N_{l}$ IoT devices in cluster *l*. Each cluster has a CH that is selected based on the residual energy on the rotational basis (see Section 4.3 for details) for a specific time duration. The CH performs the channel allocation to IoT devices for their reporting. Because we consider IoT devices to be CR compliant, they act as a SUs and access the spectrum of PUs in an opportunistic manner. A two-state on–off activity model is used to represent the channel occupancy behavior of the PUs and to predict the idle time of different channels (see Section 3.5 for details). We use the terms of IoT device, sensor node, IoTs network and WSNs interchangeably. To improve the clarity, we presents the commonly used symbols and terms in Table 1.

### 3.2. Frame Format

**Figure 2:**
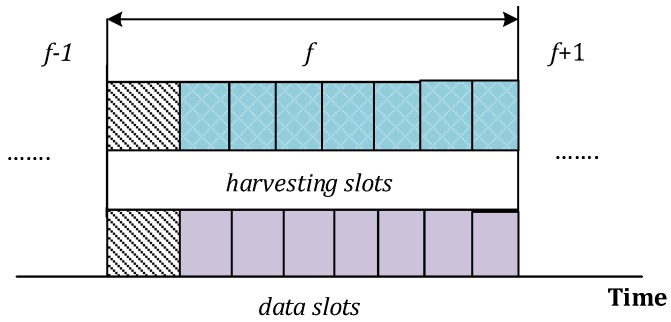
Frame format.

Figure 2 presents the frame format for energy harvesting and data transmission tasks. Because IoT devices are equipped with separate antennas for harvesting and transmission tasks, they can harvest and transmit concurrently. However, the data transmission occurs only when a CH sends a query to the desired IoT device in the reporting process. The first slot of each frame is the control slot, which assists IoT devices in exchange of control messages with RMN and the CHs during association and clustering mechanisms. For example, in the clustering method, at the beginning of each frame, all IoT devices forward their signal-to-noise ratio (SNR), PU activity and residual energy levels to their CHs. The CH performs the optimization algorithm (see Section 3.4 for details) to allocate the best possible channels to the IoT devices. This two-way exchange of control messages is done during the control slot, and we assume that it is precisely done without any synchronization hazards. Because query-based mechanism is considered for reporting, only selected IoT devices are used to govern data transmission in the reporting process, while other IoT devices remain in the idle state.

### 3.3. Energy Harvesting Model

We consider that the heterogeneous RF sources (i.e., RF sources are operating on different spectrum bands with the same or with different power levels) are available to IoT devices for EH. An IoT device *i* harvests energy from one of the best possible RF sources (see an efficient associative scheme in Section 3.2 for details about EH). The primary, as well as secondary sources, are considered for harvesting energy. However, we consider the association scheme only for primary sources. This is because we assume that primary sources use higher transmission power and they are available most of the time compared to secondary RF sources. An IoT device tunes its antenna to the frequency band of the primary RF source and starts EH. All other RF sources who are transmitting on the same frequency band will act as secondary RF sources, and the IoT device will harvest the collected energy from both sources. The RF EH rate of a sensor node *i* from primary and secondary RF source *k* can be given as follows [16]:

(1) $$E_{i,k}^{†h}=\eta P_{k}\frac{G_{k}G_{i}\lambda^{2}}{{\left(4\pi d_{i,k}\right)}^{\alpha }}\times t^{h}$$

where, $d_{i,k}$ is the distance between the harvesting source and the sensor node, *α* is the path-loss exponent and $G_{k},G_{i}$ are the antenna gains for the EH source and the sensor node, respectively. The symbol *η* indicates the harvesting efficiency and $t^{h}$ is the harvesting time. Furthermore, the † differentiates between the primary RF source (if †=1) and secondary RF source (if †=0). Considering primary and secondary RF harvesting sources, the overall harvesting energy can be described as follows:

(2) $$E_{i}^{h}=\sum_{z_{1}=1,†=1}^{\bar{P}}E_{i,z_{1}}^{†h}+\sum_{z_{2}=1,†=0}^{\bar{S}}E_{i,z_{2}}^{†h}$$

### 3.4. Energy Consumption Model

Let $E_{i,f}^{c}$ represent the energy consumed by the IoT device *i* during frame *f*. Because IoT devices are CR compliant, they can tune their antennas to the desired channel or frequency during both EH and data reporting processes. We assume that the energy consumption during channel switching is a fraction of the transmission energy. Therefore, for the sake of simplicity, we do not incorporate it into the energy consumption model. We consider that each IoT device transmits packet of fixed-size *β* bits to respective CHs during data transmission over *Z* data slots as shown in Figure 2. Because query-based reporting process (e.g., queries is generated by the CHs) is assumed, only the subset of IoT devices takes part in the reporting process, while other IoT devices remain idle during data slots.

(3) $$E_{i,f}^{c}=P_{i,f}^{tx/idle}T_{i,f}^{tx/idle}+E_{i,f}^{cir}+P_{i,f}^{ss}T_{i,f}^{ss}$$

where $E_{i,f}^{c}$ represents the overall energy consumption of an IoT device *i* during frame *f*. Because, the query-based reporting mechanism is adopted, an IoT device will operate either in the transmission mode or in the idle mode and the first term indicates the energy consumption for that possible operational mode.(i.e., if an IoT device is not taking part in the reporting process, it will remain in the idle mode with energy consumption of $P_{i,f}^{idle}$ $T_{i,f}^{idle}$). The second term quantifies the energy expenditure for electronic circuitry (e.g., filters, mixers, etc.), and the last term indicate the energy consumption due to the spectrum sensing task for the detection of PU activity to govern opportunistic channel access. Again, for simplicity, we do not incorporate energy consumption for information processing tasks.

### 3.5. Spectrum Sensing and Predicted Idle Time of Channels

As we mentioned earlier that IoT devices are CR compliant, they need to perform spectrum sensing task for opportunistic channel access. The PU signal received by an IoT device can be described using hypothesis model given in [19] as follows:

(4) $$r_{k}\left(t\right)=\left\{\begin{array}{cc} \sigma_{k}^{2}\left(t\right) & if\;H_{0}\\ x\left(t\right)h\left(t\right)+\sigma_{k}^{2}\left(t\right) & if\;H_{1} \end{array}\right.$$

where $r_{k}\left(t\right)$ is the signal received by an IoT device on channel k. The term *x*(*t*) represents the signal of a PU, *h*(*t*) is the channel response and $\sigma_{k}^{2}\left(t\right)$ shows the noise contents. For sake of simplicity, we employ the energy detector which estimates the energy of a PU signal on channel *k* as follows:

(5) $$E_{k}^{est}=\sum_{y=1}^{Y}{\left|r_{ky}\right|}^{2}$$

where *Y* is the sensed samples. if the value of $E_{k}^{est}$ is greater than the predefined threshold $\lambda_{0}$, it means that PU activity is detected on channel *k* (i.e., channel *k* is busy), and the IoT device cannot use it for transmission. However, if the value of $E_{k}^{est}$ is less than $\lambda_{0}$ (i.e., channel *k* is idle), it means PU is absent and IoT devices can use channel *k*. We use PU history to predict the idle time of the channel. Each IoT device maintains a table in which the first column shows the current status (idle or busy) of the channel *k*, and the second column indicates the average idle time $T_{avg}^{idle}$ of the channel.

(6) $$T_{k}^{prd}=\sum_{z_{0}=1}^{Z_{0}}\left(\frac{T_{avg}}{Z_{0}}\right)\cdot \pi_{k}$$

where $T_{k}^{prd}$ is the predicted idle time of channel *k*, $Z_{0}$ represents the history slots for which average idle times are maintained and $T_{avg}^{idle}$ is the average idle values of the channels. The $\pi_{k}$ is a binary variable and shows the current state (i.e., idle $\pi_{k}=1$ or busy $\pi_{k}=0$) of channel *k*.

### 3.6. Channel Capacity and Overall Throughput of a Cluster

Let $D_{i,f,k}$ be the channel capacity of an IoT device *i* during frame *f* on channel *k* computed by Shanon’s formula, and $B_{i,f,k}$ indicates the maximum number of bits that an IoT device can send on channel *k*, which can be calculated as follows:

(7) $$D_{i,f,k}=W_{k}log_{2}\left(1+SNR_{i,f,k}\right)$$

(8) $$B_{i,f,k}=D_{i,f,k}T^{prd}$$

Only those channels are useful for reporting for which $T^{prd}\geq T^{tx}$, where $T^{tx}=Q/D_{i,f,k}$ and *Q* is the size of the reporting packet. The overall throughput of IoT devices with a cluster can be computed as follows:

(9) $$R_{f,l}=\sum_{i=1}^{N}\sum_{k=1}^{M}B_{i,f,k}X_{i,k}$$

where $X_{i,k}$ represents the binary decision variable that indicates the allocation state of channel *k* to IoT device *i*. If $X_{i,k}=1$, it means that channel *k* is assigned to an IoT device *i* during frame *f* in cluster *l*, and if $X_{i,k}=0$, this means that channel *k* is not suitable for allocation.

## 4. Problem Formulation

This section provides details about the proposed association scheme, residual energy aware CH selection and throughput and QoS-aware channel allocation scheme.

### 4.1. Energy Efficient Association Mechanism for IoTs

This subsection illustrates the association scheme for IoT devices and available RF sources to maximize the overall harvested energy of the IoTs network. First, we describe the proposed four operational modes considering the characteristics of IoT devices and the nature of ambient RF sources. For each operational mode, we highlight the complexity and requirements as well. Then, we present the actual problem formulation of the association scheme for the selected operational mode.

#### Operational Modes for RF Energy Harvesting

Four operational modes are presented based on the characteristics (fixed or cognitive) of harvesting antenna of IoT devices and nature (homogeneous or heterogeneous) of ambient RF sources.

An IoT device with a fixed antenna can harvest energy only from a single band, whereas an IoT device that is equipped with the cognitive antenna can operate on multiple bands and tune itself to the desired frequency to maximize the harvesting energy. By homogeneous RF sources, we mean that all sources are transmitting on the same frequency band or belong to the same technology (e.g., LTE-A). Therefore, with homogeneous RF sources, an RF harvester only needs to tune itself to the desired frequency band for EH. However, in the case of heterogeneous RF sources, the IoT devices need an association with the best harvesting source along with the tuning of the antennas.

Table 2 highlights the proposed operational modes along with the complexity and requirements. Because IoT devices with cognitive antennas need association with RF source beside basic antenna tuning to the desired frequency band (i.e., Mode 4), its complexity is higher than simple cases (i.e., Mode 1 and Mode 2). However, an IoT device can harvest more energy from cognitive antennas. For the current article, we target the IoT devices with cognitive antennas with heterogeneous RF sources (i.e., Mode 4).

### 4.2. Association Mechanism

After the deployment of IoT devices in the sensing field, the RMN searches for ambient RF sources available in close vicinity. We assume that RMN can cover the entire sensing field and it can sense a wide range of the spectrum. The association mechanism performed by the RMN for given number of IoT devices and available RF sources can be described as follows:

1. The RMN forms a table for the location of ambient RF sources available at a given time instant. The table contains the source number and corresponding location.
2. The RMN directs a $Loc_{req}$ message to the IoT devices to provide their location.
3. The IoT devices reply with a $Loc_{res}$ message to RMN with *x*, *y* coordinates of IoT devices.
4. The RMN forms a table that contains the location information of node along with its cluster.
5. After the collection of the desired data, the RMN performs an optimization algorithm for optimal association between IoT devices and RF sources.

Mathematically, the association scheme can be formulated using the following optimization problem:

(10) $$\begin{array}{ccc} \mathrm{Maximize} & & \sum_{i=1}^{N}\sum_{j=1}^{S}E_{i,j}^{h}x_{i,j}\\ \mathrm{subject}\;\mathrm{to} & & E_{i,j}^{h}x_{i,j}\geq \zeta^{h}\;\forall i,j\\ & & x_{i,j}=\left\{0,1\right\} \end{array}$$

where $\zeta^{h}$ is the threshold for minimal harvesting energy. One good choice for $\zeta^{h}$ is that it should be greater than the energy consumed by the harvesting circuitry so that IoT devices have some input energy into the battery. To obtain the solution of Equation (10) and to ensure that the maximum amount of available energy is harvested from ambient RF sources, we use a bipartite graph-based association mechanism. Later, we extract the maximum-edge biclique graph, which provides the optimal association of IoT devices and available RF sources.

#### Bipartite Graph and Maximum Edge Biclique Graphs

**Figure 3:**
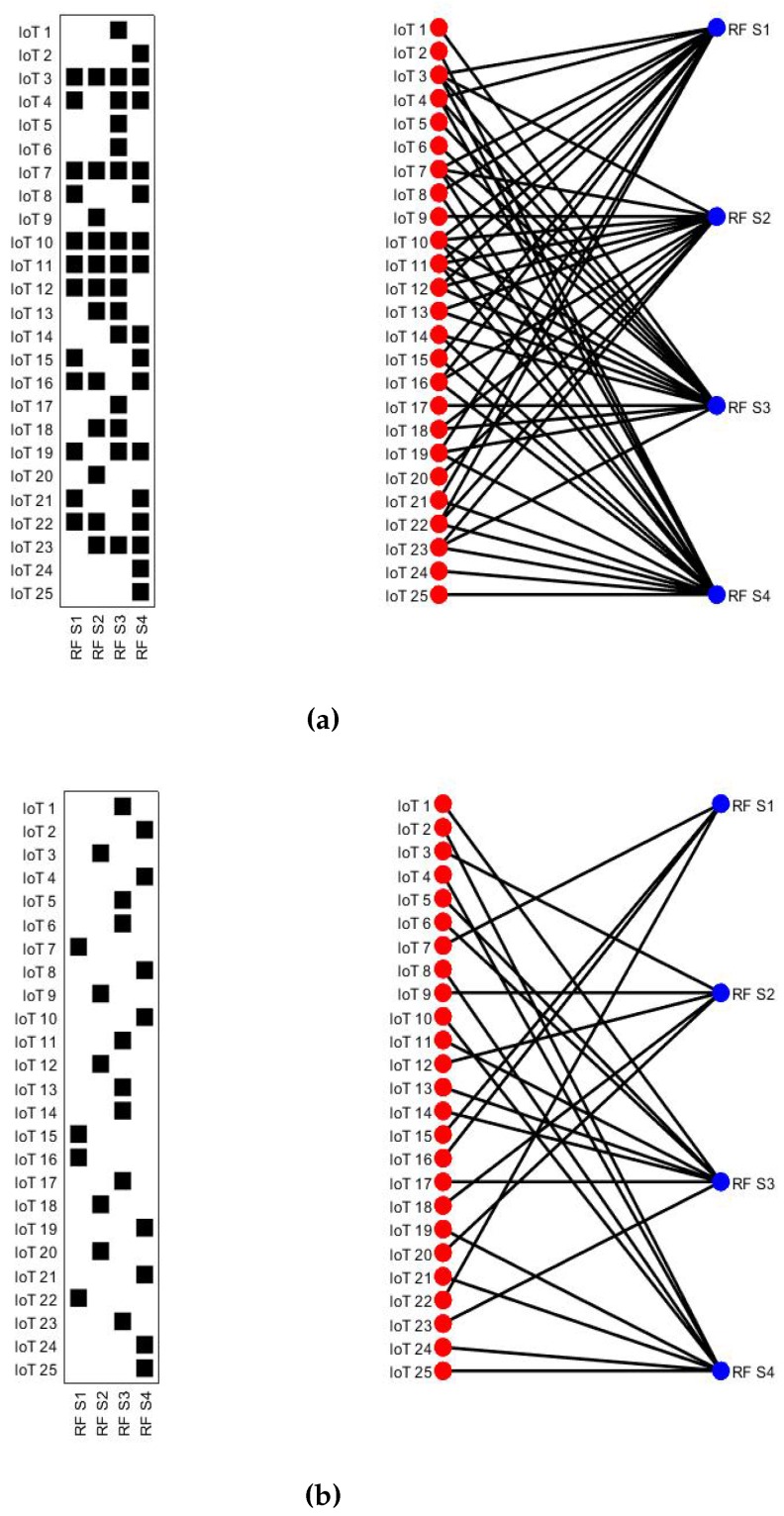
Association of IoT devices and RF sources. (**a**) Initial association using bipartite graph; (**b**) Optimal association by extracting biclique graph.

After acquiring the information from the environment about the RF sources, the RMN forms a bipartite graph. A graph *G*(*V*,*ε*) is said to be a bipartite graph if the available vertices *V* can be grouped into two disjoint sets $V_{1}$ and $V_{2}$ where $V_{1}⋃V_{2}=V$ such that any edge *ε* connects a vertex in $V_{1}$ to a vertex in $V_{2}$ [48]. Our proposed IoTs network can be represented by a bipartite graph G(N,S,ε) where the set *N* comprises of the IoT devices and the set *S* is the list of ambient RF sources. An edge $\epsilon_{i,j}$ exists between an IoT device and RF source such that *i* ∈*N* and *j*∈*S* if *i* is in the range of *j*, i.e., the IoT device can harvest energy more than a given threshold, i.e., $E_{i,j}^{h}\geq \zeta_{h}$. Figure 3a depicts the bipartite graph constructed by the RMN. Next, we construct a biclique graph $G_{0}\left(N^{\prime },S^{\prime }\right)$ in Figure 3b (see performance analysis section for details) which can be defined as follows: A biclique graph $G_{0}\left(N^{\prime },S^{\prime }\right)$ is a subgraph of a bipartite graph *G*(*N*,*S*,*ε*) induced by a pair of two disjoint subsets $N^{\prime }$ and $S^{\prime }$ such that $i\in N^{\prime }$ and $j\in S^{\prime }$ and (*i*, *j*) ∈*ε* [47]. The basic aim of the proposed association scheme is to ensure that a higher amount of ambient energy will be harvested through IoT devices. The sub-graph meeting this criterion is called the maximum edge biclique graph. Algorithm 1 extracts the maximum edge biclique graph from the given bipartite graph. The term $d^{Th}$ represents the criteria for the existence of an edge between the IoT device and ambient RF source, and $G_{0}$ is the biclique graph.

**Algorithm 1** Heuristic algorithm to extract biclique graph from bipartite graph**Require:** A bipartite graph *G*(*N*,*S*,*ε*)**Ensure:** Maximum edge biclique graph $G_{0}\left(N^{\prime },S^{\prime }\right)$ Initialize edge variable **for** (*i*: 1 to *N*) **do**  **for** (*j*: 1 to *S*) **do**   **if**
$d_{i,j}\leq d^{Th}$
**then**    Estimate $E_{i,j}^{h}$ using Equation (1)   **end if**  **end for**  Find maximum $E_{i,j}^{h}$  Find maximum $N^{\prime }\leftarrow i$ and $S^{\prime }\leftarrow j$ **end for** **return**
$G_{0}\left(N^{\prime },S^{\prime }\right)$

### 4.3. Energy Efficient Clustering Method

To achieve energy balancing among IoT devices and to perform the optimal channel allocation in the reporting process, we clustered the IoT devices into L different clusters using the *K*-means algorithm. The clustering reduces energy consumption for the reporting process (e.g., reduced distance between IoT devices and CHs) and improves overall network life-time (i.e., reduced distance minimizes transmission energy). The *K*-means clustering algorithm can be described as follows [12]:

(11) $$\bar{\Upsilon }=\sum_{l=1}^{L}\sum_{i=1}^{N}\left(\right|\rho_{i}-\rho_{l}{\left|\right)}^{2}$$

where $\left(\right|\rho_{i}-\rho_{l}{\left|\right)}^{2}$ represents the distance between node *i* and the center of corresponding cluster *l*. The parameter $\bar{\Upsilon }$ depicts the global view of the IoTs.

#### Cluster Head Selection

A CH is one of the main entities in our proposed scheme. An optimal CH selection scheme would help to achieve energy balancing. Considering the residual energy of the nodes in a particular cluster *l*, the CHs selection can be described using the following optimization problem.

(12) $$\begin{array}{ccc} \mathrm{Maximize} & & \sum_{i=1}^{N}E_{i}^{resi}x_{i}\\ \mathrm{subject}\;\mathrm{to} & & E_{i}^{resi}x_{i}\geq \zeta^{resi}\;\forall i\\ & & x_{i}=\left\{0,1\right\} \end{array}$$

where $E_{i}^{resi}$ represents the residual energy and $\zeta^{resi}$ is the threshold that ensures that the CH has significantly higher energy to perform its duties. Algorithm 2 illustrates the heuristic algorithm for CH selection in which *IE* is the intermediate energy variable for storing the residual energy of IoT devices.

**Algorithm 2** Energy pptimized cluster-head selection**Require:**  • IoT devices $N_{l}$  • Residual energy of IoT device $E_{i}^{resi}$  • Energy threshold $E^{chth}$**Ensure:** Cluster-Head selection $\phi_{1}$ Initialize selection variable $\phi_{1}\leftarrow 0$ Initialize intermediate variable $IE(1,N_{l})\leftarrow 0$ **for** (*i*: 1 to $N_{l}$) **do**  Compute residual energy of each node $E_{i}^{resi}$  **if**
$E_{i}^{resi}\geq \zeta^{resi}$
**then**   $IE\left(1,i\right)=E_{i}^{resi}$  **end if** **end for** $\phi_{1}=maxIE\left(i,i\right)$ **return**
$\phi_{1}$

### 4.4. Residual Energy and PU Activity Aware Throughput and QoS Optimized Channel Allocation

Because a query-based reporting process is considered, the CH forwards the query to the selected group of nodes, which have residual energy higher than a predefined threshold. At the beginning of each frame, the IoT devices forwards their SNR, their residual energy level, PU activity and the current state of the channel to their CHs. The CH performs channel allocation based on the optimization problem Equation (13) and forwards query to the selected nodes along the channel allocation. Then, IoT devices tune them to the desired channel for the data transmission of a fixed size reporting packet. The channel allocation problem can be formulated as follows:

(13) $$\begin{array}{ccc} \mathrm{Maximize} & & \sum_{i=1}^{N}\sum_{k=1}^{M}C_{i,k}\cdot T_{i.k}^{prd}X_{i,k}\\ \mathrm{Subject}\;\mathrm{to} & & C_{i,k}\cdot T_{i.k}^{prd}X_{i,k}\geq \zeta^{C_{min}}\;\forall i,k\\ & & \left(E_{i}^{resi}-E_{i,k}^{c}\right)X_{i,k}\geq \zeta^{remain}\\ & & \sum_{i=0}^{N}X_{i,k}\leq 1\\ & & \sum_{k=0}^{M}X_{i,k}\leq 1\\ & & X_{i,k}=\left\{0,1\right\} \end{array}$$

where, $X_{i,k}$ is a binary variable, which is either zero or one. if $X_{i,k}=1$, it means that the channel *k* is assigned to IoT device *i*. The optimization problem tries to maximize the objective function (e.g., capacity × idle time of channel) over the available channels and number of IoT devices to which the CH sends the query for reporting. There are four main constraints that ensure the optimality of the objective function. The first constraint makes sure that the selected channel meets the minimum data rate required by an IoT device. The second constraint ensures that the IoT device has the reasonable energy (i.e., above a certain threshold) to take part in the reporting process. For simplicity, we can assume that this threshold is equal to the 0.5 times the initial battery level. The Third constraint guarantees that at most one channel is assigned to one user and constraint 4 certifies that one user can occupy one channel at most. The optimization algorithm given in Equation (13) belongs to the class of the integer linear program (ILP) where the decision variable is strictly a binary variable. There are numerous ways of obtaining the solution of Equation (13). However, we use the branch and bound algorithm within Matlab function **intlinprog**. Algorithm 3 provides the step-by-step details about Matlab implementation.

**Algorithm 3** Energy and PU activity-aware throughput optimized channel allocation**Require:**  • Channel capacity C(N,M)  • Predicted idle time of channels $T^{prd}\left(N,M\right)$  • Transmission energy of IoTs $E^{tx}\left(N,M\right)$  • Residual energy of IoT device $E_{i}^{resi}$  • Transmission power of IoTs $P_{i}^{tx}$  • Capacity threshold $\zeta^{C_{min}}$  • Energy threshold $\zeta^{remain}$**Ensure:** Optimal channel allocation $\phi_{2}\left[N,M\right]$ 1. Formation of objective function $F_{obj}=C\cdot T^{prd}$ 2. Equate $F_{obj}=-F_{obj}\;for\;standard\;objective\;function$ 3. Form matrix $A_{1}$ and vector $b_{1}$ for ≥ constraint-1 and matrix $A_{2}$, vector $b_{2}$ for ≥ constraint-2 and multiply it by -1 4. Form matrix $A_{3}$ and vector $b_{3}$ for ≤ constraint-3 and $A_{4},b_{4}$ for ≤ constraint-4 5. Form standard linear non-equality constraint matrix $A=[A_{1};A_{2};A_{3};A_{4}]$ and vector $b=[b_{1};b_{2};b_{3};b_{4}]$ 6. Declare linear equality constraint matrix Aeq=[] and vector beq=[] 7. Declare lower and uper bounds: vectors *lb* and *ub* 8. Declare number of integer variables, i.e., *intvars* 9. Use function **intlinprog** to get optimal solution $\phi_{2}$ **return**
$\phi_{2}$

## 5. Performance Evaluation

This section presents the simulation results of the proposed association, clustering, CHs selection and optimal channel-allocation schemes. Furthermore, this section also compares the proposed schemes with existing ones.The performance metrics selected for evaluation are the (1) average harvesting energy; (2) successful reporting probability (SRP); and (3) number of alive nodes in the network. The performance of the proposed scheme is compared by varying the distance (meters), number of frames, PU activity, number of available channels, primary harvesting sources, secondary harvesting sources and IoT devices. The Monte Carlo simulation model is adopted to get average results for 500 different iterations. Matlab’15 is used to acquire simulation results. Although the given simulation results are equally valid for more generic cases with the higher density of IoT devices, we present the simulation results for an environment where N=25 IoT devices are operating in a geographical area of size 1000 × 1000 m^2^. For the association mechanism, we use a heuristic algorithm for IoT devices and plot the bipartite and maximum edge biclique graphs. The CHs are selected on a rotational basis using the residual energy of the nodes as selection criteria. Four heterogeneous RF sources are located at ($X_{1}^{s}=350,Y_{1}^{s}=350)$, $\left(X_{1}^{s}=350,Y_{1}^{s}=750\right)$, $\left(X_{1}^{s}=750,Y_{1}^{s}=350\right)$ and $\left(X_{1}^{s}=750,Y_{1}^{s}=750\right)$. For average idle-time computation, the PU history is maintained for Z=10 frames and the average value is computed over these 10 frames. We assign weight is in descending order from recent to old frames. The noise power spectral density is −110 dBm/Hz and the bandwidth of the channels *W* is selected from a range of 1∼8 MHz. The rest of the important simulation parameters are shown in Table 3.

Figure 3 shows IoT devices and primary RF harvesting sources operating at [700 900 1800 2100] MHz , respectively (for proof of concept, we consider only N=25 IoT devices and $\bar{P}=4$ primary RF sources. However, our results are equally valid even for a very high density of IoT devices and available RF sources). The transmission power of each primary RF source is considered to be 46 dBm. The distance of IoT devices varies from 5∼500 m within the sensing field. Figure 3a depicts the bipartite graph where each edge between IoT device and RF source indicates that an IoT device can harvest energy from that particular source. However, this bipartite graph is showing the many-many relationship. For optimal harvesting, we find a biclique graph using proposed heuristic Algorithm 1 which converts a many-many relationship into a one-many relationship where each edge shows the optimal relationship between the IoT device and RF source as shown in Figure 3b. For example, the IoT devices i=3,i=9,i=12,i=18,i=20 are associated with RF harvesting source j=2 to maximize the overall harvesting energy of the IoTs network.

**Figure 4:**
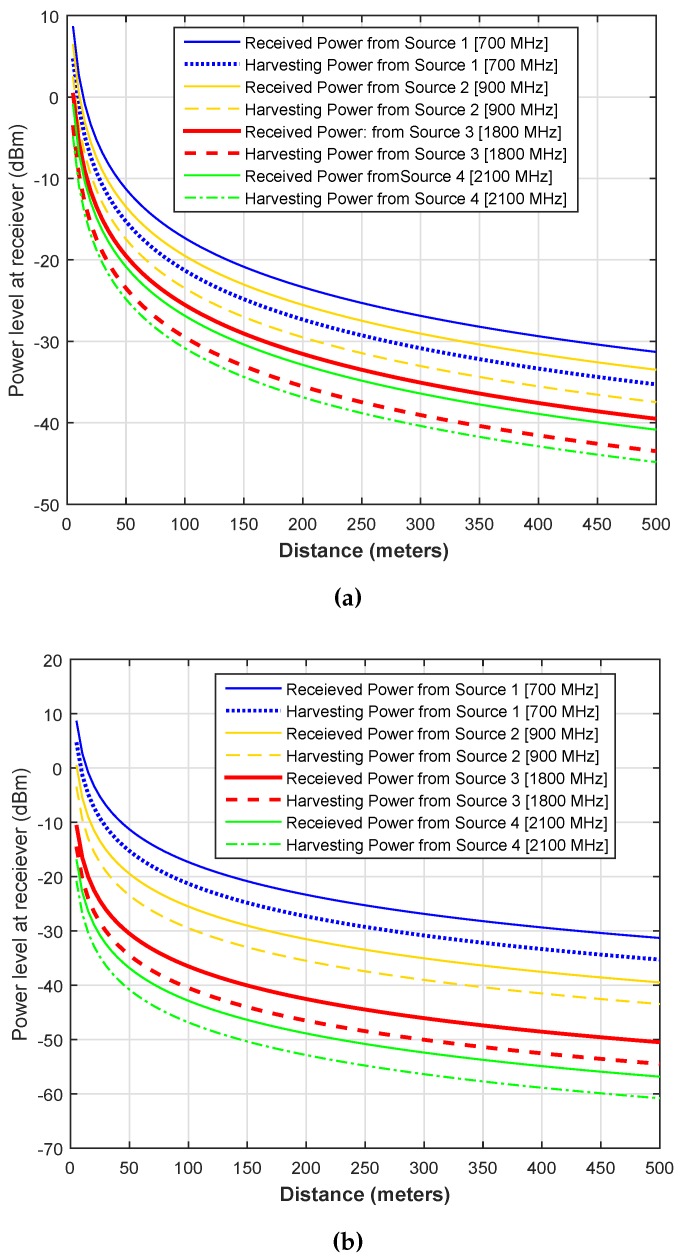
Received and harvested powers at receiver. (**a**) Same power and link gains of RF sources; (**b**) Different power and link gains of RF sources.

Figure 4 illustrates the average received and harvesting powers from different RF sources operating at [700 900 1800 2100] MHz, respectively. The variations in received and harvesting powers are plotted by varying the distance of nodes from 0∼500 m. The rest of the simulation parameters are the same as that mentioned for Figure 3. In this simulation result, we try to depict the heterogeneity of RF sources in terms of operating frequency bands and transmission powers. In Figure 4a, we use the same power of 46 dBm, whereas in Figure 4b, we use the power of [46 40 35 30] dBm for [700 900 1800 2100] MHz sources, respectively. It is clear from the two results that the highest energy can be harvested from that primary source for at 700 MHz source with 46 dBm of power and harvesting energy decreases as we move to the higher spectrum. Furthermore, the overall harvesting energy decreases with a decrease in the transmission power or an increase in the distance. One important thing is the difference (i.e., the difference between solid and dashed or dotted lines) between the received power and the harvesting power. This is due to the lower harvesting efficiency *η* of the receiver.

**Figure 5:**
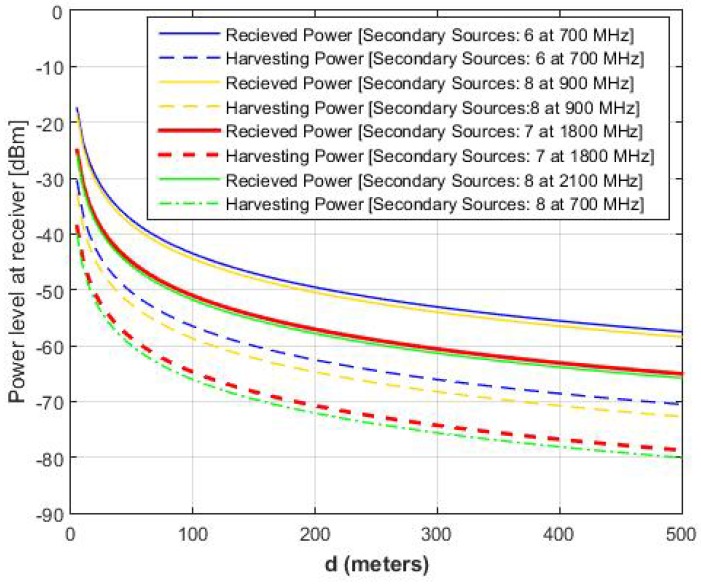
Probability of successful reporting for different node-classification schemes.

**Figure 6:**
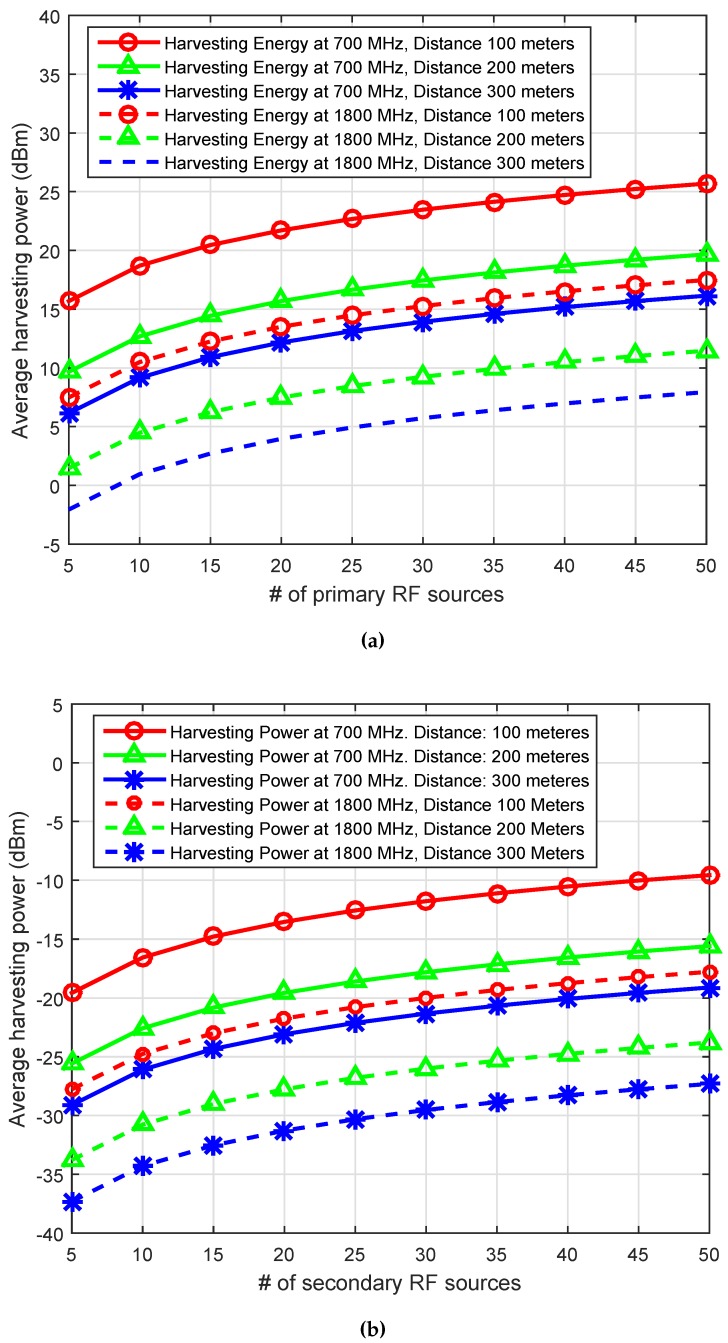
Received and harvesting power at receiver. (**a**) Same power and link gains of RF sources; (**b**) Different power and link gains of RF sources.

Similarly, Figure 5 illustrate the received and harvesting power from different secondary RF sources at [700 900 1800 2100] MHz with $\bar{S}=\left[6\;8\;7\;8\right]$. The result shows the decline in the harvesting efficiency with an increase in the distance. Compared to the harvesting power of the primary RF sources shown in Figure 4, the average harvesting power from secondary RF sources is 20 dBm lower than the harvesting power from primary RF sources. To provide further insight into the harvesting power or energy, we vary the number of primary RF sources (i.e., $\bar{P}$ = 5∼50 (Figure 6a)) and secondary RF sources (i.e., $\bar{S}$ = 5∼50 (Figure 6b)), and we plot the average received and the harvesting powers at frequency of [700 1800] MHz and the distance of [100 200 300] m. Both results show an increase in the harvesting power with an the increase in harvesting sources. However, there is a sharp decline with the increase of the distance between the RF source and IoT devices.

**Figure 7:**
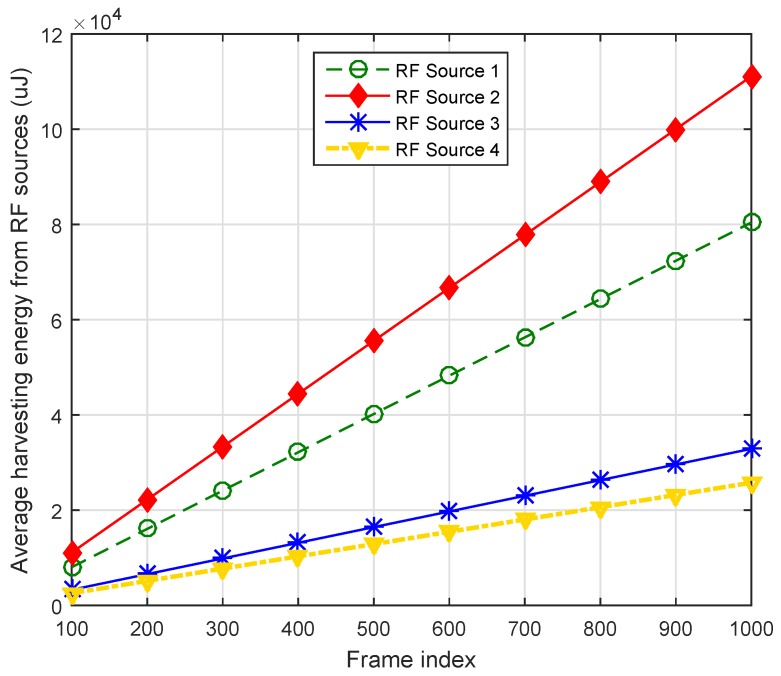
Average harvesting energy from RF sources.

**Figure 8:**
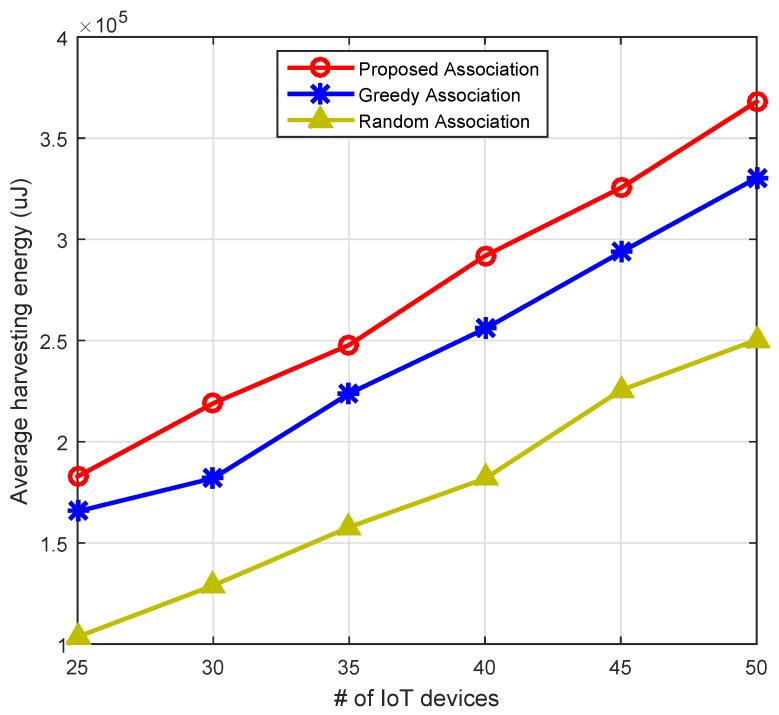
Comparison based on average harvesting energy.

Figure 7 illustrates accumulated harvesting energies from different primary sources across different frame indexes. It is clear from the simulation result that accumulative harvesting shows a linear increase with the increase of the frames. The IoT devices belong to a cluster that is near the RF source transmitting at a 700 MHz band with power 46 dBm which are harvesting more powers as compared to the other IoT devices that belong to the cluster of RF sources operating at [900 1800 2100] MHz, respectively. Figure 8 compares the proposed association mechanism with greedy and random approaches. The comparison is drawn based on the average harvesting energy from primary and secondary RF sources. It is clear from the result that the proposed heuristic algorithm of association optimized the RF harvesting energy compared to the greedy and random association mechanisms. For example, with 40 IoT devices, the proposed association mechanism harvests 12.4% and 37.69% higher energy, respectively, as compared to the greedy and random association approaches. Hence, our proposed biclique graph-based association scheme is the most suitable for IoT devices.

**Figure 9:**
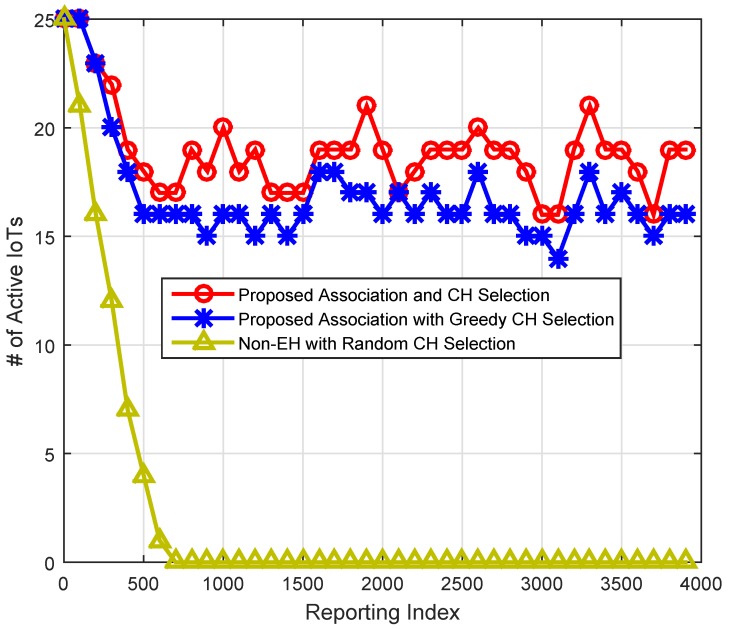
Comparison based on alive IoTs across different frames.

Figure 9 illustrates the advantage of the energy efficient association and energy-aware CH selection scheme. We plot the number of active IoTs at various reporting indexes. *An IoT device is said to be active if its residual energy is equal or more than 50% of its initial energy*. There are two noticeable things about the pattern of the results. First, the proposed scheme shows better performance as compared to the other schemes. Second, the proposed EH and association schemes switch the nodes between an active and nonactive state; hence, the network will remain alive for a longer duration. With the proposed CH selection mechanism, there are a higher number of active IoTs at any given frame index as compared to the greedy and random CH selection schemes. For example, consider frame index 500; the proposed scheme shows 12% and 77.88% higher alive IoTs, respectively, compared to the greedy and random CH selection schemes. Hence, with the proposed association and CH selection mechanisms, the network can remain alive longer and more IoTs can participate in the reporting process.

**Figure 10:**
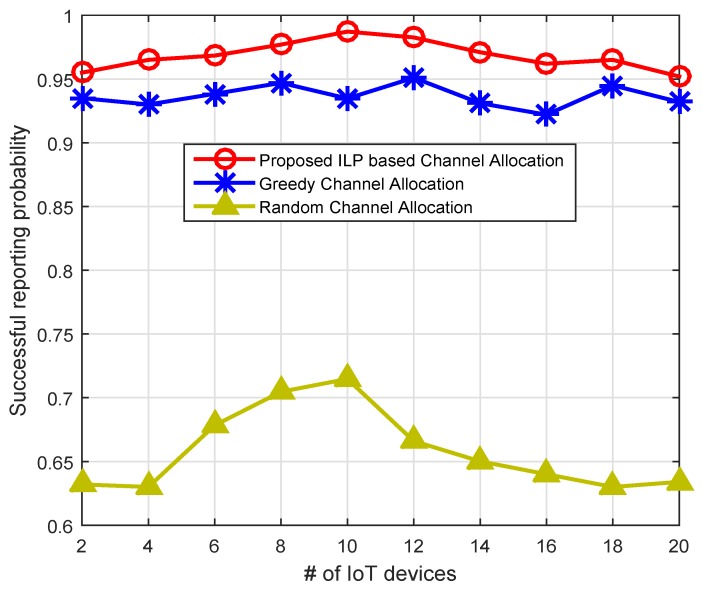
Comparison based on successful reporting probability across number of IoT devices.

**Figure 11:**
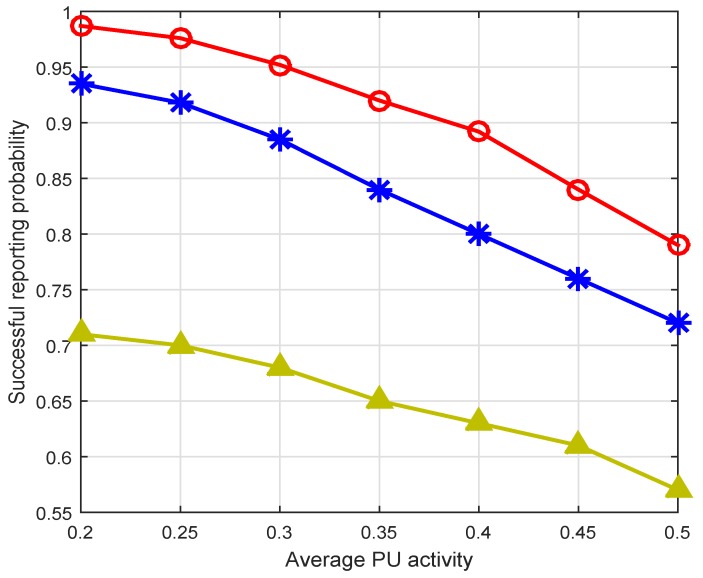
Comparison based on successful reporting probability across PU activity.

Figure 10 shows the successful reporting probability (SRP) using our residual energy and PU activity-aware throughput and QoS optimization scheme in comparison with the greedy and random channel allocation schemes. A reporting is considered to be successful if the residual energy of the node is higher than the 50% of its initial energy, and the channel satisfies the minimal QoS criteria. The PU activity also reduces SRP if it arrives in the middle of the reporting process. The greedy channel allocation (GCA) scheme selects IoT device *i* and allocates channel *k*, which has the highest value of the objective function as shown in Equation (13). After channel allocation, the GCA marks that IoT device and channel as assigned and removes them from the pool of channels and IoT devices. Then, the same procedure is repeated for the rest of the nodes until all channels are allocated to IoT devices. The random channel allocation (RCA) scheme randomly picks the IoT device *i* with probability $1/\bar{N}$ and channel k with probability $1/\bar{M}$, and channel allocation is performed for reporting process. Similarly, other IoT devices will get channel with the same procedure. The proposed energy and PU activity-aware throughput and QoS optimization scheme shows significantly better successful reporting probability compared to GCA and RCA. For example, with 10 IoT devices, the proposed scheme shows 27.6% and 5.31% better SRP as compared to the RCA and GCA schemes. Similarly, in Figure 11, we plot the SRP against different PU activity (i.e., different PU arrival rate). The SRP declines exponentially with the increase of the PU activity for all three schemes. However, the proposed channel allocation scheme shows up to an 8% and 30% better SRP compared to the GCA and RCA schemes.

## 6. Conclusions

This paper presents an IoTs framework that addresses three issues including energy efficiency, energy balancing and QoS for IoT devices. To achieve energy efficiency, an innovative RF energy-harvesting scheme is presented where IoT devices are associated with the best possible RF sources to maximize the overall harvested energy by these IoT devices. For energy balancing, the IoT devices in close proximity are grouped together in a cluster, and the IoT device with highest residual energy is selected as a CH that allocates the channels to the IoT devices considering their desired QoS. Each of these three presented schemes are compared with the greedy and random approaches. To evaluate energy efficiency and energy balancing, comparisons are drawn based on the average harvested energy across different frames and the number of alive IoTs at different frames and reporting indexes. For both of these cases, the proposed schemes shows significantly higher efficiency compared to the greedy and random approaches. For QoS support, comparisons are drawn based on the successful reporting probability and average throughput by varying IoT devices, PU activity and number of available channels. Again, the proposed ILP-based channel-allocation scheme shows the highest throughput and better reporting probability compared to the greedy and random approaches. Hence, the proposed schemes are well suited for the IoTs framework to address aforementioned challenges. In the future, we will extend the work to develop a scheme that will select RF sources and perform channel assignment to IoT devices to optimize harvesting energy and QoS under single optimization problem. 

## Figures and Tables

**Table 1:** Symbols and notations.

Symbols	Meaning
$F_{1},F_{2}$	Objective functions
N	Number of IoT devices
M	Number of channels
L	Number of RF sources
*i*	Subscript of IoT devices
*j*	Subscript of RF sources
*k*	Subscript of RF channels
$E_{i,k}^{†h}$	harvested energy of IoT device *i* from RF source *k*
$E^{h_{pr}}$	harvested energy from primary RF source
$E^{h_{sec}}$	harvested energy from secondary RF source
$P_{k}$	Transmission power of RF source *k*
*η*	Harvesting efficiency
$t^{h}$	harvesting time
$G_{i}$	Link gain of an IoT device
$G_{k}$	Link gain of an RF source
$d_{i,k}$	Distance between RF source and IoT device
*α*	Pathloss exponent
*λ*	Wavelength
$\lambda_{0}$	Threshold for energy detector in spectrum-sensing
*Q*	Size of reporting packet
$X_{i,k}$	Binary decision variable for channel allocation
$x_{i}$	Binary decision variable for CH selection

**Table 2:** Operational modes.

Modes	IoTs	RF Sources	Complexity	Requirements
1	Fixed Antenna	Homogeneous	Very Low	Switch on
2	Fixed Antenna	Heterogeneous	Very Low	Switch on
3	Cognitive Antenna	Homogeneous	Low	Tunning
4	Cognitive Antenna	Heterogeneous	Medium	Tuning & clustering

**Table 3:** Simulation parameters.

Parameters	Values
Power of primary RF source	46 dBm
Power of secondary RF source	16 dBm
Noise power spectral density	−110 dBm/Hz
Channels	10∼30
PU activity	0.0∼0.6
Sensing interval	0.1 msec
Modulation scheme	MQAM
Constellation size	4
Constellation size	4
Size of reporting packet *Q*	10 KB
Transmission Power of IoTs $\left(P^{tx}\right)$	10 dBm
Spectrum sensing Power of IoTs $\left(P^{ss}\right)$	4 dBm
Energy consumption of circuitry $\left(E^{cir}\right)$	50 nJ/bit
History partitions	3
Weights [$\omega_{1},\omega_{2},\omega_{3}$]	[0.6 0.25 0.15]
